# The ‘community’ in community case management of childhood illnesses in Malawi

**DOI:** 10.3402/gha.v9.29177

**Published:** 2016-01-27

**Authors:** Wanga Z. Zembe-Mkabile, Debra Jackson, David Sanders, Donela Besada, Karen Daniels, Texas Zamasiya, Tanya Doherty

**Affiliations:** 1 Health Systems Research Unit, South African Medical Research Council, Cape Town, South Africa; 2 School of Public Health, University of the Western Cape, Bellville, South Africa; 3 Knowledge Management & Implementation Research Unit, UNICEF, New York, USA; 4 School of Child and Adolescent Health, Faculty of Health Sciences, University of Cape Town, Cape Town, South Africa; 5 UNICEF, Malawi Country Office, Lilongwe, Malawi; 6 School of Public Health, University of the Witwatersrand, Johannesburg, South Africa

**Keywords:** community health workers, community involvement, Malawi

## Abstract

**Background:**

Malawi has achieved a remarkable feat in reducing its under-5 mortality in time to meet its MDG 4 target despite high levels of poverty, low female literacy rates, recurrent economic crises, a severe shortage of human resources for health, and poor health infrastructure. The country's community-based delivery platform (largely headed by Health Surveillance Assistants, or HSAs) has been well established since the 1960s, although their tasks and responsibilities have evolved from surveillance to health promotion and prevention, and more recently to include curative services. However, the role of and the form that community involvement takes in community-based service delivery in Malawi is unclear.

**Design:**

A qualitative rapid appraisal approach was utilised to explore the role of community involvement in the HSA programme in Malawi to better understand how the various community providers intersect to support the delivery of integrated community case management by HSAs. Twelve focus group discussions and 10 individual interviews were conducted with HSAs, HSA supervisors, mothers, members of village health committees (VHCs), senior Ministry of Health officials, district health teams, and implementing partners.

**Results:**

Our findings reveal that HSAs are often deployed to areas outside of their village of residence as communities are not involved in selecting their own HSAs in Malawi. Despite this lack of involvement in selection, the high acceptance of the HSAs by community members and community accountability structures such as VHCs provide the programme with legitimacy and credibility.

**Conclusions:**

This study provides insight into how community involvement plays out in the context of a government-managed professionalised community service delivery platform. It points to the need for further research to look at the impact of removing the role of HSA selection and deployment from the community and placing it at the central level.

## Introduction

It has been argued that the ideal form of community participation should both mobilise communities to carry out health programmes, as well as increase ‘people's control over the social, political, economic, and environmental factors determining their health’ (1). However, it is widely acknowledged that true community participation is rarely achieved (1).

The recruitment and selection of community health workers (CHWs) is seen as a key component of community participation and as an important step in ensuring the success and proper functioning of any community health strategy (2). Recruitment of CHWs is defined as ‘how and from where a CHW is identified, selected, and assigned to a community, including selection criteria and processes’ (3). The recruitment of CHWs by and from within their communities of residence is regarded as best practice and contributes to increased retention of CHWs, programme quality, and greater accountability of CHWs and the programmes they implement (2).

When the Millennium Development Goals were first established in 1990, Malawi had high rates of infant and under-5 mortality at 143 and 242 per 1,000 live births, respectively (4). In 2015 under-5 mortality was estimated to have dropped to 64 per 1,000 live births (4), enabling Malawi to achieve its MDG 4 (to reduce under-5 child mortality by two-thirds between 1990 and 2015) target. Malawi's success in achieving a dramatic reduction in under-5 mortality has occurred despite considerable contextual challenges including high rates of poverty, low female literacy rates, recurrent economic crises, a severe shortage of human resources for health, and poor health system infrastructure (5, 6).

Child survival gains in Malawi have been attributed to a number of policies and programmes that have been implemented to reduce child mortality. Amongst these has been the decentralisation of child health care services to the community level through the implementation of the integrated Community Case Management of Childhood Illnesses (iCCM) strategy in 2009 (7). iCCM's main objective is to manage and treat children suffering from uncomplicated common childhood illnesses such as diarrhoea, malaria, pneumonia, and newborn sepsis using simple, proven high impact interventions (8).

Community-level health services in Malawi comprise three hierarchical layers. At the top there is the village development committee (VDC) which is made up of representatives from all the villages in a district. The function of this structure is to provide oversight to the village health committees (VHCs) as well as to address community development issues at a wider scale beyond health. VHCs provide oversight to the Health Surveillance Assistants (HSAs) who are front-line CHWs delivering community health services in villages across Malawi. VHCs act as a link between the community and HSAs – they support the work of HSAs by referring sick children to them, and assist HSAs in community mobilisation activities (9).

HSAs are responsible for delivering the iCCM package of child survival interventions primarily in hard-to-reach areas, in all 28 districts of Malawi. Hard-to-reach areas are defined as rural areas which are difficult to access due to long distances (more than 5 km from the nearest health facility) and other physical barriers such as impassable rivers and mountains. HSAs have been an integral part of the Malawi health system for nearly 50 years. They were originally introduced in the 1960s as temporary ‘Smallpox Vaccinators’ and then as ‘Cholera Assistants’ in the 1970s, and mainly played the role of environmental health monitors in the 1990s, becoming formally established as government-salaried workers in 1995 (7). To qualify for recruitment, HSAs are required to have 12 years of schooling and are remunerated with about US$110 per month. iCCM in Malawi was preceded by the Essential Health Package (EHP) which was introduced in 2004 as a strategy to prioritise and strengthen community participation and the free delivery of community health services (10). Child-specific conditions targeted in the EHP include malaria, diarrhoea, pneumonia, and eye infections amongst other conditions. The prominence of HSAs began to grow with the introduction of the EHP, thus by the time iCCM was introduced in 2009, HSAs were already providing child-focused interventions, although these did not include curative functions.

Studies investigating iCCM in Malawi have reported on quality of care (11), programme scale up (7), and the perceptions of health workers and managers of the programme (12). Much less has been published about the community involvement aspects of iCCM. Gaps in knowledge exist with respect to how iCCM has been implemented at the community level, as well as the role that community participation has had in the programme's acceptance and uptake. Such knowledge is important because community participation has long been identified as a cornerstone of primary health care and as critical to improving health outcomes (13).

Against this backdrop, the Malawi HSA programme is an interesting case study in how community participation plays out in a large government-managed programme. HSAs are entrenched in the health system, holding multiple responsibilities including promotive, preventive, and curative work. HSAs divide their time between iCCM-focused activities in the village clinic, and preventive and promotive activities; in some villages this translates to 3 days spent on iCCM where community members bring their children to the village clinic, and the other 2 days is spent in the community; in some communities the HSA opens the village clinic in the mornings for iCCM cases and spends the afternoons in the community.

The HSA delivery platform is not a small non-governmental project; it is a large government-funded programme and the HSAs are themselves salaried employees of the Government of Malawi (GoM), entirely managed within the country's health system. One of the consequences of the central management of HSAs by the GoM is that their selection and deployment follows a top-down approach which excludes communities. This results in HSAs, who are deployed to rural areas, often travelling far to reach their catchment populations. A lot quality assurance survey undertaken in 2013 revealed that only 36% of hard-to-reach villages had an HSA living there (14).

This paper explores community involvement in the delivery of iCCM by HSAs in hard-to-reach areas in Malawi, with a particular focus on the role of community involvement in the recruitment and deployment of HSAs, as well as the role of community-level accountability structures such as VHCs. In this study we define the ‘community’ as people who reside in the same catchment where community health services are implemented.

## Methods

This qualitative study was a substudy of a large multicountry evaluation of the Catalytic Initiative (CI) Integrated Health Systems Strengthening (IHSS) programme of support to six countries in Africa: Malawi, Ethiopia, Ghana, Mozambique, Mali, and Niger between 2007 and 2013 (15).

The evaluation in the six countries took place in 2012 and 2013. As we needed to gather data in a short period of time, we evaluated the CI/IHSS programme as a case study utilising rapid appraisal (RA) methods. The RA approach uses a less structured format for data collection in order to obtain the required information in a timely and cost-effective manner (16, 17). It is often used in cases where there are time and budget constraints to conducting a study or evaluating a programme. Although RA methods limit the ability to generalise findings to the larger population, they are useful in formative evaluations and provide a snapshot of what is happening in a given context. In this way, the RA approach provides a starting point in the study of any phenomenon.

In this study the RA approach was used because it enabled the collection of data over a period of 8 working days in Malawi, and it formed part of and strengthened the data triangulation process because the larger evaluation utilised desktop review methods, secondary quantitative data analysis, and qualitative research techniques (6).

### Participants and sampling

In advance of the country visit we sent a proposed list of interviewees, identified through a desk review of programme reports and documents, to the United Nations Children's Fund (UNICEF) country team, who then assisted with prescheduling appointments. In compiling this list we gave consideration to gaining as wide a range of opinion as possible so as to ensure a fair representation of how community involvement in the delivery of iCCM was experienced in Malawi.

The research team made contact with potential participants by email for the Ministry of Health (MoH) and implementing partner categories and the UNICEF country office made contact with potential district and community-level participants by email and phone. The only reason given for non-participation amongst senior MoH and implementing partner participants was if the person was travelling at the time of fieldwork and would not be available for an interview. It is difficult to determine the extent of non-participation amongst health facility and community-level participants as they were invited to be present at the facility for an interview by the UNICEF staff. We do know that in these hard-to-reach rural communities subsistence farming is practiced so women may have stayed away because they were busy or because it was hard-to-reach the clinic. Furthermore we always reinforced at the beginning of each interview that the interview was voluntary and no one left after the offer to leave was made.

It is also important to bear in mind that in this context, poor people are vulnerable, so they are unlikely to be frank about why they would decline to participate. If for example they were unhappy with the programme, then they would perhaps just not show up, but they wouldn't necessarily admit that this was why.

Individual interviews and focus group discussions (FGDs) were held with several categories of participants: UNICEF staff, implementing partners, Malawi Ministry of Health staff, District Health Team members, HSA supervisors, nurses in health facilities, HSAs, VHC members, and mothers in three districts (Lilongwe, Kasungu, and Mzimba). Table 1 provides a summary of all of the participants interviewed.

**Table 1:** Summary of participants

	Participant category	Number of interviews
Individual interviews	HSAs	3 (male)
	Health Centre Medical Officers	2 (male)
	Ministry of Health	4 (1 female, 3 male)
	Implementing partners	1 (male)
Focus groups	HSAs (2–8 per group)	2 (majority male)
	Community members (mothers) (5–10 per group)	2 (all women)
	Village health committee	1 (males)
	District Health Management Team	2 (1 with 3 people all male; 1 with 9 people 7 male, 2 female)
	UNICEF Country office	1 (4 people – 2 female and 2 male)
	Implementing partners	4 (2 with 2 people, 1 female, 1 male; 1 with 3 men; 1 with 2 female and 1 male)

### Data collection

A country visit to Malawi took place over 8 days in August 2013. TD, DJ, DS, DB, and KD conducted the interviews. Both individual interviews and FGDs were conducted as was deemed appropriate for the kinds of experiences we hoped to explore with the different participant categories. Policymakers engage with health programmes individually, so their individual response is important. Other participant categories such as HSAs and mothers are more likely to have a collective response, because they talk to each other, and there are group norms around what is appropriate when dealing with sick children. So understanding those group norms was important to understanding how HSAs and mothers as a group responded to the programme (Table 1).

In general, interviews with senior MoH staff, implementing partners, health centre staff, HSA supervisors, and some of the HSAs were conducted in English as these participants were comfortable conversing in this language. The researchers were all English speaking therefore for interviews with non-English speakers (largely HSA and beneficiary interviews) the researchers conducted the interviews in the presence of a local person, who acted as an interpreter and helped interviewers to establish rapport with the participants.

The researchers obtained informed consent from participants for interviews. Different semi-structured interview guides were used for each respondent type to guide the discussion around the experiences of implementing iCCM. At different times during fieldwork the team met to discuss the interviews and revise topic guides accordingly, thus the development of guides was an iterative process.

All interviews took place either at the offices of the interviewees, at a district office, health centre, or in the communities (e.g. in a community hall) and they were all audio recorded.

### Data analysis

Data were transcribed and checked against the original recording to ensure accuracy. During each interview, field notes were written to capture the context and compliment the audio recordings. All data were analysed using manual content analysis methods (18). Together with some of the co-authors, the lead author read through each of the transcripts, noted initial thoughts, and began manifest coding of the data. Coding began with an inductive analysis of the data whereby meaning units were identified in the transcripts and transformed to condensed meaning units. The condensed meaning units were then summarised into codes. The codes were grouped together into categories that were then further transformed into major themes. An example of the analysis process is provided in Table 2. WZM and TD discussed this first phase of the analysis, and once there was agreement on the codes, categories, and themes generated from this analysis, the same process was used in the analysis of the remaining transcripts. Furthermore, during data analysis, WZM had feedback and discussion sessions with the rest of the co-authors to validate, discuss, and interpret the themes. Several different themes emerged such as those related to HSA supervision, HSA performance, community involvement, training, and supply chain management. For this paper we chose categories and themes related to community involvement as the manuscript's analytic angle.

**Table 2:** Examples of the analysis process

Interview	Meaning units	Condensed meaning units	Codes	Categories
Mothers’ interview	‘… they say if it is middle of the night they know that they can't find the HSA here, so its either the husband or other family members start walking to the facility’	HSA who lives outside community is not available all the time for sick children	HSAs not always available	HSAs deployed outside areas of residence
Mothers’ interview	‘… some are saying that the problem is that there is no house for the HSA to live, that is the challenge. They saying he wants to stay here but the problem is accommodation …’	HSA wants to stay in catchment area but no accommodation	HSA accommodation a challenge	HSAs deployed outside areas of residence
Village health committee	‘… we discuss some issues like the importance of going to under five clinics with the children who are under 5. We discuss about the importance of going to the antenatal clinics for the pregnant mothers. We discuss about the use of a protected source of water in the communities to prevent other … diseases’	Village Health Committee members hold community meetings to discuss importance of attending under five clinics	Village health committee promote	HSAs work Village Health Committee roles

### Ethics

This study received ethical approval from the South African Medical Research Council (EC026-9/2012). Approval for the in-country data collection was also granted by the Malawi UNICEF country office and the Malawi Ministry of Health.

Before each interview, the interviewees explained the purpose of the interview in detail and as far as possible ensured that participants understood what agreeing to participate in the study would mean. When necessary we used the services of a translator to explain our research aim and the consent process. Participants who agreed to participate either signed a consent form or gave their consent verbally. All participants except for UNICEF staff and implementing partners were given payments of 1,300 Kwacha (about US$5) each to compensate them for transport and lunch costs.

### Findings

There were five main themes related to community involvement that emanated from the analysis; these were:

1. Community participation in the recruitment and selection of HSAs
2. Community involvement in the provision of accommodation for HSAs deployed outside their areas of residence
3. Community members’ views on the impact of HSA deployment outside their areas of residence
4. Community members’ appreciation for HSAs
5. Community overseers – the role of VHCs in iCCM

#### Community participation in the recruitment and selection of HSAs

Between 1960 and 1990, when HSAs in Malawi mainly worked as cholera assistants and on environmental health outreach, they were selected by their communities, and/or by community leaders, with the guidance and involvement of health workers. During this period they therefore worked in their village of residence. As one informant explained:

They were elected by the chief around this area. (VHC member)

However, senior MoH officials reported that this changed approximately 4 years prior to our study, when HSAs began to incorporate curative child health functions into their roles in the form of the iCCM package of interventions. As HSAs became more professionalised, the MoH opted to recruit and select HSAs at the district level, and finally with the introduction of iCCM, HSA recruitment was conducted at the national level, and consequently it was no longer guaranteed that HSAs would work in the same areas they came from. Senior officials suggested that recruitment was centralised due to concerns about fairness as well as the difficulty of finding persons who met the educational requirements for HSA selection, because literacy in the rural areas is low.

… the trend has changed, previously, they were selected at district level … providing and ensuring that they would be coming from their own areas so they can serve the people better. Five years ago I think there were these issues to do with human rights where government put in measures to say let us explore opportunities for everybody other than just limiting people to be picked from wherever they want to choose. With that pressure you know the Ministry of Health was pressed enough to abandon the previous system of deploying or recruiting people from the area. (Senior MoH official)

Now these things are advertised at national level and in one way or the other also affected the way we are deploying and keeping these people in their respective catchment areas because people from one district would be recruited and employed in another district. (Senior MoH official)

Informants suggested that changes to the selection and deployment of HSAs have affected HSA retention and their ability to carry out their duties, as they are not able to be available for the communities they serve during the expected hours in a week as they are meant to.

#### Community involvement in the provision of accommodation for HSAs deployed outside their areas of residence

HSAs are required to live in the area to which they have been deployed. However, this is a challenge because the hard-to-reach areas to which HSAs are deployed are often without adequate accommodation. Communities are encouraged to help provide accommodation for HSAs who do not come from their areas. This is done by constructing village clinics with an attached living area for the HSA.

One of the factors linked to the lack of adequate housing for HSAs is that villagers are often the ones expected to construct accommodation for HSAs. As one official explained:

What we have done through the district councils, the district assemblies, they did the district management team to ensure mobilization of communities to at least construct some housing for accommodation for the HSA. (Senior MoH Official)

However, it is clear from our interviews that there are challenges with this approach. For one, as pointed out by key informants, communities do not have the required resources to build proper sturdy housing structures. In some cases non-government organisations build village clinics, and in other cases the government attempts to mitigate this challenge by subsidising the cost of building these houses through the provision of basic building materials:

… support is given from what we call the Local Development Fund from the District Council, especially iron sheets because those are the most expensive things … [and also] some cement is provided. These are basically smaller makeshift housing within the same house, one side is an attachment what we call the village clinic, so you have in each clinic one side would be the clinic and the other side would be the HSA residing inside the house. (senior MoH official)

However, very few houses have been built, despite government and or donor assistance. A 2013 survey found that only 36% of hard-to-reach villages had an HSA living there (14).

#### Community members’ views on the impact of HSA deployment outside their areas of residence

The construction of village clinics that are able to double up as accommodation for HSAs is important, as living outside the catchment area negatively affects the work of HSAs. Interviews with community members revealed their own preference for HSAs to live in the communities they work in. Narratives relayed by community members suggested that time spent travelling into catchment areas has resulted in HSAs being unavailable to see sick children at all times; caregivers are therefore compelled to take their children to a health centre, often miles away from where they live.

… So if they (HSAs) say they will be staying here in the community they (community members) feel they can be effective any time when a child is sick, [so] despite that they know the challenge of accommodation but they will be happy if the HSAs will be residing here …. (VHC member).

… they say if the HSA was to move within this community, they feel because then anytime their child is sick he can come and see to them. Some are saying that the problem is that there is no house for the HSA to live, that is the challenge. They saying he wants to stay here but the problem is accommodation …. (Mothers’ FGD)

Furthermore, community informants reported that HSAs who reside outside the areas they work in often came into the village clinic late and left early as a result of the long distances they had to travel, often on foot, from their villages to the catchment area.

… they [are] saying the HSA comes in the morning, sometimes he comes a bit late, yes, so they say that is an issue, in the morning he is here [late] because they come and wait a few minutes or even a few hours yes … they are saying they would love it if the HSA can be here the whole day and not knock off earlier before the knock off time. Most of them come around 3 or 4 [pm], so that is one of their concerns. (Mothers’ FGD)

the problem as I have already said I'm not staying within the community; I am living a little bit far from here so for me, I don't have means of transport … sometimes I walk … that's why I have said [the bicycle] it's unreliable, ja, I've got it but most of the times I come here on foot. (HSA)

#### Community members’ appreciation for HSAs

Despite the logistical concerns related to HSA placements, community members interviewed for this study were very appreciative of the role that HSAs played in health service provision and the fact that their presence in communities made care more accessible.

Our own observations in the field reflect community members’ appreciation of HSAs. Some of the HSAs we met extended themselves beyond the scope of their tasks. Furthermore, some HSAs had drawings of maps of their communities in their village clinics where they had plotted every home in the village:

they saying when a child is sick, they bring the child … that's what they're saying, of course, she continued to say before the village clinic was open they had problems … during the raining season they cannot pass but since the village clinic is open, they bring the children here because it's closer. (Mothers’ FGD)

she [says] even if maybe the child is sick any other day apart from Mondays, Wednesdays, and Fridays, when they come the HSA assists … very well when they come they [are] given the help they need …. (Mothers’ FGD)

#### Community overseers: the role of VHCs in iCCM

VHCs represent another layer of community service delivery in Malawi and provide support for HSAs operating in village clinics. Their involvement in community health work dates back to the 1990s when the MoF established VHCs to promote primary health care activities such as planning for environmental health services including water and sanitation and disseminating preventive health messages to their communities. VHCs work on a voluntary basis and are selected by chiefs (with help from HSAs) in each community where there is a village clinic or HSA working in that area. Field interviews demonstrated that VHC presence was critical to the overall effectiveness of HSAs as they facilitated community participation, governance, and ownership. We also learnt from interviews that VHCs engage in community outreach and health promotion activities. Additionally, they provide support and assistance to HSAs by referring caregivers with sick children to them and by encouraging community members to make use of HSA services:

… [we have] meetings with the communities at least, maybe 3 or 4 times a month with communities … with different communities, yes … we discuss some issues like the importance of going to under-5 clinics with the children who are under 5. We discuss about the importance of going to the antenatal clinics for the pregnant mothers. We discuss about the use of a protected source of water in the communities to prevent other … diseases. (VHC Member)

Composed of an equal number of males and females, during fieldwork we experienced VHCs as being very visible at village clinics where they seemed to play an oversight role over HSAs ensuring that they came in to work, and promoted the services of HSAs in their communities. In this way VHCs in this setting were an example of accountability made visible; of community engagement and community-level governance in action, with their presence legitimising and bolstering community confidence in HSAs:

the village health committee is strong because they are all ten members, they come and they assist the issues here at the clinic … the committee has ten [members], 5 are women, 5 are men. Normally they divide [visits to the village clinics] amongst themselves, two people come. For the week, two members of the community, the other week another two members, because they want to make sure that the HSA is around … first he [the VHC member] makes sure during the clinic day that they [VHC members] are there to see how the HSA is doing … and interact with the community to tell them the goodness of taking their [HSA] advice. (VHC member)

As pointed out in interviews, their oversight role is especially exercised in the management of the drug supply that HSAs keep in their village clinics. Here, VHCs have shared responsibility for the wooden medicines box and hold one key to the box while HSAs keep the other. Respondents explained that this mechanism was put in place to prevent abuse of the drugs (by HSAs), as well as to deter potential theft.

These people you know usually, they have a wooden drug box. The wooden box has two lockable keys. One key is kept by a member of the VHC. Why? You know HSA can see we are required to assist you at any time, even at night, or during the day. One to avoid … abuse, you know when I see a child come any other day, other than the scheduled clinic days, a message is sent on mobile phone because most of the people have mobile phones, it says have patient and bring the other key, help so I can assist children, at night it is the same, for the safety of the HSA, people will just come and attack him, so a member of the VHC is informed and has one key to make sure that we have safety and proper support. (Senior MoH official)

However, our findings show that the practice of VHCs controlling access to the drug box is not strictly adhered to in all village clinics. In some village clinics flexibility is allowed to ensure that caregivers are able to access supplies and medicines for their children in the event that a VHC or the keys are not available.

In one case, an HSA explained that he kept a separate small supply of emergency drugs in case he does not have access to the key or the VHC is not available to open the box.

I keep an emergency supply (outside the box). (HSA interview)

In some of the interviews we learnt that in some communities VHCs were required to leave the key behind when unavailable or traveling outside the catchment area.

We are advised they must leave the key behind …. (HSA interview)

## Discussion

Community involvement in supporting HSA delivery of iCCM in Malawi consists of several layers including VDCs and VHCs. Each of these cadres fulfils certain roles and functions in supporting HSAs in the provision of basic primary health care within hard-to-reach areas.

Malawi has made great strides in developing a cadre of CHWs (HSAs) who are salaried and employed by the government as part of the national health workforce and are capacitated to diagnose and treat the common diseases of childhood. However, as part of the process of scaling up and increasing the scope of HSA work, the selection process has been removed from communities and is now a national-level process requiring formally advertising positions at district and national levels, as with standard government employment processes.

Community participation is fundamental to the selection process of CHWs. Literature highlights two important aspects of the selection process: ‘(1) that CHWs should be chosen from the communities they will serve and (2) that communities should have a say in the selection of their CHWs’ (1) (p 18). Evidence suggests that CHWs who are selected by and deployed to their own communities have a greater impact on health outcomes, utilisation, and health promotion (19). In addition, CHWs that are from the catchment areas they serve are said to have greater retention rates, and to be more likely to be invested in the health outcomes of the populations they serve (2).

As depicted in our findings, in Malawi HSAs are neither (necessarily) deployed to the communities they come from, nor are the communities involved in their selection. Literature on the recruitment and selection of CHWs shows that, although not ideal, it is not all that unusual for CHWs to be deployed outside the areas they reside in, nor is the exclusion of communities in the selection of CHWs (20). Furthermore, it seems that the lack of community participation in the selection and deployment of CHWs is widespread globally. Indeed, Lehmann and Sanders conclude that ‘participatory selection processes remain an ideal that is relatively rarely practised, particularly in large-scale programmes’ (1).

Evidence generated from studies across different settings suggests that when community participation is fully realised, it facilitates the acceptance and appreciation of CHWs by community members (19). Although our findings in this paper indicate that community members appreciated the role and presence of HSAs in their communities regardless of whether they lived in the community or not, it is uncertain whether this sentiment will remain in the long term if the selection process of HSAs continues to exclude community engagement. Some qualitative findings from other studies undertaken in Malawi suggest that communities are not always willing to build village clinics and HSA accommodation even if the building material is made available (21). In one case, several catchment areas in one of the districts in Malawi were tasked with building village clinics and housing for their HSAs (who reside outside the areas) while drugs and other necessary equipment to run the clinics were provided for by a donor. However, after 2 years, only about a third of the clinics/houses had been built, and the rest of the catchment areas had not built any structures (21). As such, the issue of HSAs deployment and how this is viewed and perceived by communities is not straightforward.

Furthermore, lack of facilities such as schools and opportunities for trade act as a disincentive for HSAs to relocate to areas outside their own communities. An added complication in the Malawi context is the targeting of hard-to-reach areas for iCCM, as by definition such areas offer little in the way of trade opportunities, and thus have less appeal for prospective HSAs.

The professionalisation of CHWs may also affect how this cadre of workers is perceived (and received) by communities. Participants in a small qualitative study in South Africa suggested that although community members enjoyed the enhanced health care access as a result of having a CHW who was recruited from and lived in their community, they also questioned the elevated authority of someone they regarded as being like themselves (22).

In the literature great emphasis is placed on the need for CHWs to be accountable to their communities, and VHCs are seen as an important institutionalised form of community participation responsible for guiding and managing the work of CHWs (1). VHCs when operating properly are said to facilitate community participation and provide legitimacy, guidance, and governance in community health programmes (23). Although our findings suggest that the role of VHC members is mainly that of oversight, other research findings indicate that the place they occupy in the community health system of Malawi is not straightforward. Despite positive feedback and a strong sense of empowerment by VHC members highlighted through our interviews, research reported by others revealed that VHCs do not seem to have much power in the implementation of iCCM and are essentially presented as subordinate to HSAs.

## Strengths and Limitations

Our study had some limitations. As this was an RA of the implementation of iCCM by HSAs in Malawi, our ability to undertake an in-depth exploration of the study topic was limited. The use of the RA approach in this study meant that we were not able to explore issues in great depth while in the field because our interaction with participants was once off and we had limited scope to use constant comparison while in the field. As a consequence of our brief time in the field, we were not able to do member checks and we did not return to the field to report back; thus we have not been able to establish the validity of our findings.

We also depended on UNICEF for the selection of some participants and this means that we could have interviewed people who were inclined to speak favourably about the programme. However, a particular strength of the study was that it was conducted by an experienced team of researchers, some of whom had previously conducted studies in Malawi, and the participants included a wide range of stakeholders from national government to mothers receiving services from HSAs.

## Conclusion

This study has explored the role that community involvement plays in the Malawi community delivery platform, and in particular, how it intersects with the work of HSAs. It contributes to the existing evidence base about the extent to which community participation and involvement affects the acceptance and ability of CHWs to do their work effectively in low- and middle-income country contexts.

Our findings show that the work of HSAs in Malawi is comprehensive as it incorporates treatment preventive and promotive aspects. Thus this CHW model combines ‘new’ task-shifting and ‘old’ CHW duties. Both the latter (preventive and promotive) require active community participation.

Furthermore, although community involvement and participation operate in a somewhat unconventional way in this setting in that communities are not involved in selecting their HSAs, and HSAs are often deployed to areas outside where they live, communities, through structures such as the VHCs, still play an important role in the oversight of HSAs, and in giving the programme legitimacy and credibility.

Further research is needed to tease out the complexities raised by contradictory evidence on the role of VHCs and perceptions of their usefulness, as well as the precise impact of removing the role of HSA selection from communities on CHW effectiveness.
